# Right ventricular volumes vs. right ventricular ejection fraction are more powerful independent predictors of survival in patients with severe ischemic cardiomyopathy

**DOI:** 10.1186/1532-429X-14-S1-P3

**Published:** 2012-02-01

**Authors:** Noreen Nazir, Rory Hachamovitch, Zoran B Popovic, Scott D Flamm, Thomas Marwick, Deborah Kwon

**Affiliations:** 1 Cleveland Clinic, Cleveland, OH, USA

## Background

Right ventricular ejection fraction (RVEF) has been shown to be an independent predictor of mortality after myocardial infarction. However, the predictive value of right ventricluar (RV) assessment in patients with severe ischemic cardiomyopathy (ICM) is unknown.

## Purpose

In patients with severe ICM, we sought to assess the association of RVEF, RV end systolic and diastolic volumes (RV ESVi/RV ESDVi), right ventricular systolic pressure (RVSP) with outcomes in severe ICM.

## Methods

450 patients with > 70% stenosis in ≥1 epicardial coronary artery (75% men, median age 63 years, median LV ejection fraction (EF) 22 %, median ESVi 106ml, median RV EF 45%. Median RVESVi 37.5ml) underwent delayed hyperenhancement-MRI between 2002-2007 (Siemens 1.5-T scanner, Erlangen, Germany) between 2003-2007. CMR evaluation included long and short axis assessment of LV function on balanced steady state free precession images along with assessment of myocardial scar (on phase-sensitive inversion recovery DHE-CMR sequence ~ 10-20 minutes after injection of 0.2 mmol/kg of Gadolinium dimenglumine). Cox proportional hazards survival modeling, using a primary end-point of all-cause mortality), was used to risk-adjust comparisons. Echocardiograms performed within 60 days were analyzed, and RVSP was assessed as described by American Society of Echocardiogram guidelines.

## Results

Results: Over a follow-up of up to 9 years[mean 5.75 years], 186 deaths occurred. Survival analysis revealed that after adjusting for left ventricular scar burden, left ventricular ESVi, subsequent revascularization, sex, age, mitral valve procedures, RVSP (χ2 7.37, p = 0.007), RVESVi (χ2 4.81, p = 0.028), and RVEDVi (χ2 4.11, p = 0.042) were independent predictors of mortality. See Table 1. However, RV EF (χ2 3.22, p = 0.073) was not an independent predictor of mortality.

**Table 1:** 

Variable	Chi-square	p=value
RVSP	7.37	0.007
RVESVi	4.81	0.028
RVEDVi	4.11	0.043
RV EF	3.22	0.073

## Conclusions

Conclusions: RV volumes and RVSP, provide independent, incremental prognostic value in patients with severe ICM. RV EF did not provide independent prognostic value.

## Funding

None.

